# Waist Circumference Coupled with Either HDL-C or TG Can Be Used as a Diagnostic Marker for Metabolic Syndrome in Chinese Women with Polycystic Ovary Syndrome

**DOI:** 10.1155/2018/6102085

**Published:** 2018-09-10

**Authors:** Yan Sun, Wenxiang Wang, Qi Shen, Shengrong Du, Yiwei Guo, Fei He, Wenchang Zhang

**Affiliations:** ^1^Reproductive Medicine Center, Fujian Provincial Maternity and Children's Hospital, Affiliated Hospital of Fujian Medical University, Fuzhou, Fujian, China; ^2^Fujian Province Key Laboratory of Environment and Health, Fujian Medical University, Fuzhou, Fujian, China; ^3^Department of Health Inspection and Quarantine, School of Public Health, Fujian Medical University, Fuzhou, Fujian, China

## Abstract

Although quite a few polycystic ovary syndrome (PCOS) patients suffering from metabolic syndrome (MS) have been reported in previous studies, no reliable and early diagnostic biomarkers for MS in PCOS patients have yet been identified. To identify early and reliable diagnostic biomarkers for MS in Chinese women with PCOS, a total of 401 patients (200 PCOS patients and 201 controls) were enrolled in our present study. All of the subjects were examined for anthropometric (weight, waist circumference, blood pressure, etc.) and biochemical (fasting glucose, serum lipid indices, total testosterone, etc.) parameters. Our results showed that the prevalence of MS in the PCOS patients (20.50%) was 6.8-fold higher (*P* < 0.05) than that in the controls (2.99%). Nearly 71.0% of the PCOS patients had at least one component of MS, of which dyslipidemia was the most prevalent. Furthermore, within the PCOS group, the prevalence of MS increased with increasing age and body mass index (BMI). Logistic analysis indicated that BMI, triglycerides (TG), high-density lipoprotein cholesterol (HDL-C), hypertension, and fasting glucose were significantly associated with the presence of MS in PCOS patients. Analysis of the ability of the potential diagnostic biomarkers to indicate MS in PCOS patients showed that the PPV, NPV, specificity, sensitivity, and Youden's index for waist circumference (WC) coupled with HDL-C were 59.68%, 97.10%, 84.28%, 90.24%, and 74.52, respectively, and those for WC coupled with TG were 93.33%, 92.35%, 98.74%, 68.29%, and 67.03%, respectively. ROC curve analysis showed that the areas under the ROC curves (AUCs) for WC coupled with HDL-C and for WC coupled with TG were 0.882 and 0.901, respectively. Our present study demonstrates that WC coupled with either HDL-C or TG can be used as a relatively early and reliable diagnostic biomarker for MS in Chinese PCOS patients.

## 1. Introduction

Polycystic ovary syndrome (PCOS) is a common gynecological endocrine disease with an incidence of 5–10% in premenopausal women and is one of the common endocrine causes of infertility in women of childbearing age [1–3]. Women with PCOS also have a higher risk of gynecological cancers [4–6]. Previous studies have demonstrated that PCOS is associated with multiple metabolic abnormalities, including obesity, dyslipidemia, and impaired glucose tolerance, which are also components of the metabolic syndrome (MS) [7]. Therefore, PCOS increasingly affects both the reproductive system and metabolic system in relation to MS [8].

The metabolic syndrome can severely affect health; its manifestations include obesity, hyperinsulinemia, insulin resistance (IR), abnormal lipid metabolism, high blood pressure, and many other metabolic diseases [9, 10]. Similarly, PCOS is also considered a risk factor for type 2 diabetes mellitus and cerebrovascular disease, suggesting the importance of identifying early biomarkers for MS in PCOS patients. Due to differences in race, lifestyle, diet, genetic factors, and MS diagnostic criteria, the data reported in previous studies on the prevalence of MS and its components in PCOS patients are not consistent. Therefore, no reliable biomarkers for screening PCOS patients for MS risk have yet been identified [11–13]. In 2005, the International Diabetes Federation (IDF) announced a unified global definition of MS, with central obesity being an essential diagnostic criterion [12].

The main purpose of this study was not to explore the causal inference but to compare the incidence of MS and its components (based on the IDF MS diagnostic criteria [12]) between PCOS patients and controls in Chinese women and then to identify relatively early and reliable diagnostic biomarkers for MS in Chinese PCOS patients for early intervention.

## 2. Materials and Methods

### 2.1. Subjects

We consecutively recruited 200 PCOS patients (20.0–38.0 years old) who were diagnosed between January 2016 and December 2016 by the gynecologists at Fujian Provincial Maternity and Children's Hospital. The control group consisted of 201 women (20.0–39.0 years old) who were consecutively recruited from women receiving physical examinations during the same period. The women in the control group had regular menstrual cycles and did not have clinical manifestations of hyperandrogenism or hyperandrogenemia. The study was conducted under the approval of the Ethics Committee of the Foundation of Fujian Provincial Maternity and Children's Hospital, and informed consent was obtained from each participant.

### 2.2. Diagnostic Criteria

PCOS was diagnosed based on the diagnostic criteria of the Rotterdam workshop sponsored by the American Society for Reproductive Medicine [11]. The diagnosis of MS was based on the IDF definition, including the core criterion of waist circumference (WC) ≥ 80 cm for Chinese women and the presence of any two of the following four conditions: (i) elevated triglycerides (TG) (>1.7 mmol/L) or current treatment for this lipid abnormality, (ii) decreased high-density lipoprotein cholesterol (HDL-C) (<1.29 mmol/L) or current treatment for this lipid abnormality, (iii) systolic blood pressure (SBP) ≥ 130 mmHg or diastolic blood pressure (DBP) ≥ 85 mmHg or current treatment for hypertension after diagnosis with this disease, and (iv) fasting glucose (FG) ≥ 5.6 mmol/L or diagnosis of diabetes [14].

### 2.3. Study Protocol

A uniformly designed information registration form was used to register all the enrolled patients. The anthropometric parameters included height, weight, WC, hip circumference, blood pressure, hirsutism score, and acne score, and BMI (weight (kg)/height (m^2^)) and waist-to-hip ratio (WHR: waist (cm)/hip (cm)) were calculated. For weight evaluation, the Asia-Pacific region diagnostic criteria from the World Health Organization (WHO) were used: a BMI < 18.5 kg/m^2^ is considered to be low body weight, a BMI of 18.5–22.9 kg/m^2^ indicates normal weight, a BMI of 23.0–24.9 kg/m^2^ indicates overweight, and a BMI ≥ 25 kg/m^2^ indicates obesity [15]. Investigators have been trained regularly, and accuracy and precision are used to evaluate the standardization of measurement.

Fasting venous blood samples were drawn on days 2–5 of the menstrual cycle (or for amenorrheic patients, when a B-mode ultrasound was not able to detect dominant follicles). The levels of total testosterone (TT) and dehydroepiandrosterone sulfate (DHEAS) were determined by immunochemiluminescence. The FG, fasting insulin (FI), total cholesterol (TC), TG, HDL-C, apolipoprotein A (Apo-A), and Apo-B levels were also analyzed in fasting venous blood samples. The enzymatic endpoint method was used to determine the levels of TC and TG. The HDL-C level was measured by a direct method using polyethylene glycol-pretreated enzymes. The Friedewald formula was used to calculate low-density lipoprotein cholesterol (LDL-C). The immunoturbidimetric method was used to measure Apo-A and Apo-B. FG was determined by the glucose oxidase method using an automatic biochemical analyzer (Abbott ARCHITECT ci16200, Abbott Park, IL, USA). The immunochemiluminescence method was used to determine FI. The FI, homeostasis model assessment of insulin resistance (HOMA-IR = (FG (mmol/L) × FI (*μ*U/mL))/22.5), and quantitative insulin sensitivity check index (QUICKI = 1/(log10 FG (mg/dL) + log10 FI (*μ*U/mL))) were used to assess the degree of IR [16, 17]. The standard of each index was used to check the accuracy of the method, and all samples were run in duplicate in a single assay to reduce interassay variation.

### 2.4. Statistical Analyses

The IBM SPSS 20.0 software package was used for the statistical analysis. The Kolmogorov-Smirnov test was performed to test the normal distribution of all the continuous variables. Because the continuous variables did not show normal distributions, the data were reported as the median and interquartile ranges (25th–75th percentiles). The differences between the groups were evaluated by the Mann–Whitney *U* test. Logistic regression analysis was performed to assess the relative contributions of various factors (age, BMI, WC, TG, HDL-C, FG, and hypertension) to the diagnosis of MS in PCOS patients. Pearson's chi-square test was used to analyze the differences in the constituent ratios between the MS and non-MS groups. The relative risk odds ratios (ORs) and 95% confidence intervals (CIs) between the MS and non-MS groups of PCOS patients were also calculated. The positive predictive value (PPV), negative predictive value (NPV), specificity, sensitivity, and Youden's index were used as evaluation indices for screening for MS. Receiver operating characteristic (ROC) curve analysis was used to generate AUC values to evaluate the predictive ability of MS characteristics for the diagnosis of MS in PCOS patients (the highest Youden's index was set as the cutoff value). A *P* value of less than 0.05 indicated that a difference was statistically significant.

## 3. Results

### 3.1. Patient Characteristics

The anthropometric and biochemical parameters are shown in Table 1. Compared with the controls, the PCOS patients had elevated levels of WC, TT, DHEAS, TG, TC, LDL-C, Apo-B, DBP, FG, FI, and HOMA-IR (*P* < 0.05) and decreased levels of HDL-C, Apo-A, and QUICKI (*P* < 0.05).

### 3.2. Prevalence of MS and MS Components in PCOS Patients

Of the 200 PCOS patients, 41 (20.50%) met the IDF MS diagnostic criteria. This prevalence was significantly higher than that of the control group (2.99%). Of the PCOS patients, 29.0% did not have any MS components, 34.5% had one MS component, 16.0% had two MS components, 16.5% had three MS components, 3.0% had four MS components, and 1.0% had five MS components. A detailed analysis of the MS components revealed that the percentage of dyslipidemia (represented by abnormal levels of HDL-C and TG) was the most prevalent component of metabolic disorders: the occurrence of reduced HDL-C was 51.0% and the occurrence of high TG was 19.50%. The second most prevalent component was high blood pressure, which was seen in 13.50% of the patients. The patients with abnormal blood glucose comprised only 5.50% of the patient group. The occurrence of at least one metabolic disorder component in the PCOS group was 1.5 times greater than that in the control group (Table 2).

**Figure 1:**
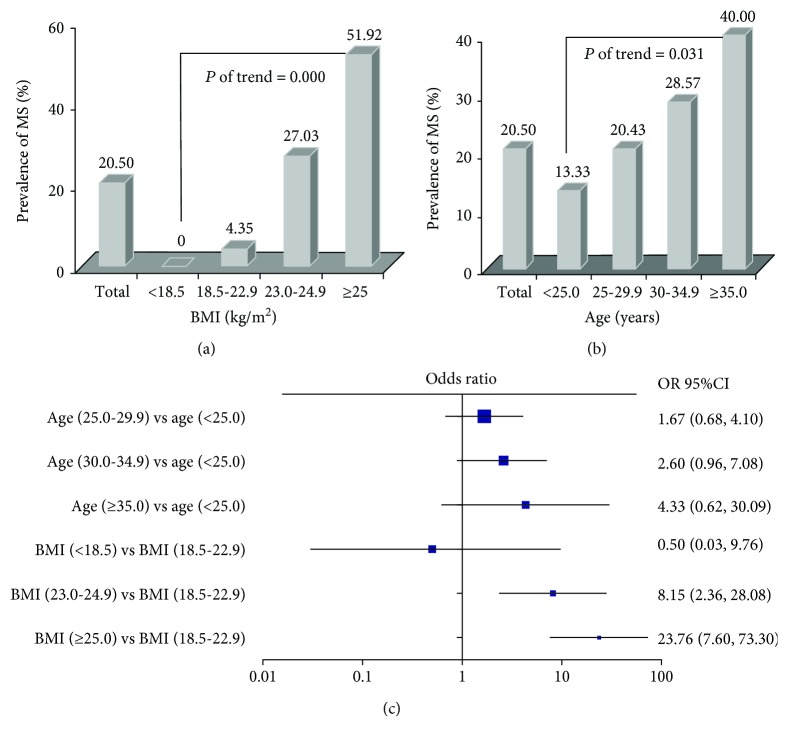
The effect of BMI (a) and age (b) on the prevalence of MS and the association of age and BMI (c) with the prevalence of MS in PCOS patients (*n* = 200).

Moreover, an analysis of the prevalence of MS in PCOS patients by age and BMI was also performed in our present study. The result showed that MS was present in the PCOS patients younger than 25 years of age and that the prevalence of MS gradually increased with age (*P* < 0.05). The results also showed that the prevalence of MS gradually increased with increasing BMI (Figure 1(a)). When the groups of patients aged 25.0–29.9 years, 30.0–34.9 years, and ≥35.0 years were compared to the group of PCOS patients aged <25 years, there were no statistically significant differences in the ratios of PCOS patients suffering from MS (Figure 1(b)). A detailed analysis of the changes in the occurrence of MS components with increasing age revealed no clear increasing trends (Table 3). There was no occurrence of MS in underweight PCOS patients. The risk of developing MS in the overweight group was 8-fold greater than the risk in the normal weight group (OR, 8.15; 95% CI, 2.36–28.08). The risk of developing MS in the obese group was 23-fold greater than the risk in the normal-weight group (OR, 23.76; 95% CI, 7.60–74.30) (Figure 1(c), Table 4).

### 3.3. ROC Curve Analysis to Assess Potential Diagnostic Markers for MS in PCOS Patients

**Figure 2:**
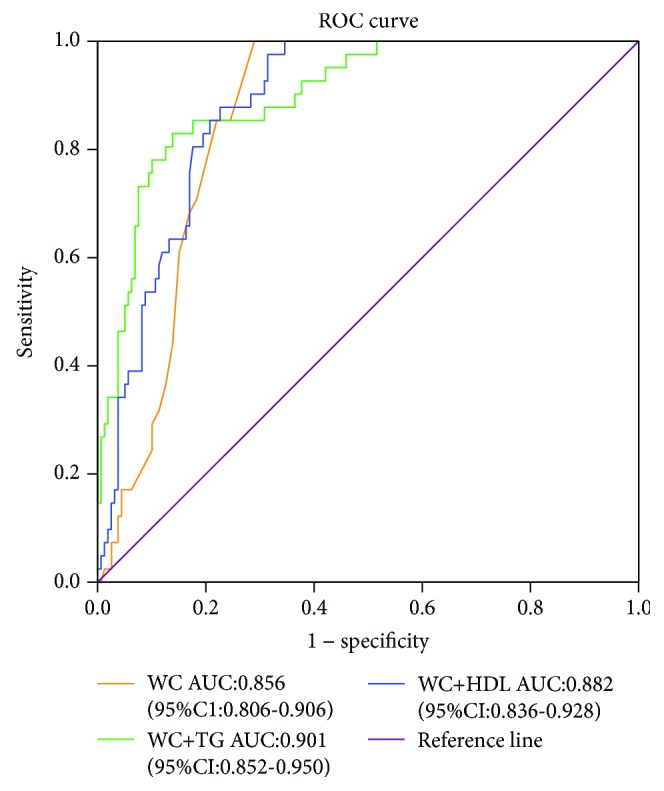
Receiver operating characteristic curve for assessing the screening ability of waist circumference (WC), WC coupled with triglycerides (TG), and WC coupled with high-density lipoprotein cholesterol (HDL-C) in PCOS patients (*n* = 200). AUC: the areas under the ROC curve. Reference line: chance line.

According to the IDF MS diagnostic criteria, central obesity is a prerequisite for the diagnosis of MS. In this study, we first used PPV, NPV, specificity, sensitivity, and Youden's index as indices for the evaluation of WC coupled with different MS components as diagnostic biomarkers for MS. The potential biomarker of WC coupled with HDL-C was analyzed. Youden's index was 74.52, the PPV was 59.68%, and the NPV was 97.10%. The potential biomarker of WC coupled with TG was also analyzed. Youden's index was 67.03, the PPV was 93.33%, and the NPV was 92.35%. In addition, the sensitivity was 68.29%. In addition, the potential biomarker of WC coupled with blood pressure was analyzed. Youden's index was 48.15. However, the PPV was the highest of all combinations evaluated. Finally, the potential biomarker of WC coupled with FG was analyzed. This potential biomarker had the smallest Youden's index (16.44) and the lowest sensitivity (17.07%) of all combinations evaluated (Table 5). Furthermore, we compared the usefulness of WC coupled with HDL-C and WC coupled with TG for screening the risk of MS in the PCOS patients. The results showed that the areas under the ROC curves (AUCs) for WC coupled with HDL-C and for WC coupled with TG were 0.882 (95% CI = 0.836–0.928) and 0.901 (95% CI = 0.852–0.950), respectively (Figure 2). There was no statistically significant difference between the two groups (Figure 2). Therefore, WC coupled with HDL-C or WC coupled with TG can be identified as relatively reliable diagnostic biomarkers for MS in PCOS patients.

## 4. Discussion

In this study, we found that the prevalence of MS in Chinese PCOS patients is significantly higher than that in the controls. Within the PCOS group, the prevalence of MS increased with increased age and BMI. Furthermore, using PPV, NPV, specificity, sensitivity, and Youden's index as evaluation indices to evaluate the potential diagnostic ability of WC coupled with different MS components, we demonstrated that WC coupled with HDL-C and WC coupled with TG are relatively reliable diagnostic biomarkers for MS in PCOS patients.

The concept of MS was first proposed by Reaven who defined it as a cluster of clinical syndromes characterized by the simultaneous presence of multiple metabolic abnormalities [17]. PCOS is a heterogeneous disease that is often accompanied by IR, hyperinsulinemia, and dyslipidemia [18]. Since many of the clinical characteristics of PCOS and MS overlap and are internally correlated, PCOS has been proposed to be a natural model for MS [19].

In our present study, we found that there were significant differences in lipid metabolism and IR between the PCOS group and the control group. This finding is consistent with those in previous studies by other investigators, as it has been shown that hyperlipidemia can promote IR through inhibiting insulin and glucose delivery to target cells and by reducing the use of glucose in peripheral blood [20]. In addition, IR can also cause hyperlipidemia by increasing the synthesis and secretion of very-low-density lipoprotein cholesterol (VLDL) and TG in the liver and by reducing the clearance of VLDL and TG [21]. Therefore, hyperlipidemia is an important factor leading to IR, and IR can also increase the probability of lipid metabolism disorders. Lipid metabolism disorders in PCOS patients mainly manifest as increased levels of TG, TC, and LDL-C and decreased HDL-C levels [22]. In addition, there is a reduction in the Apo-A level and an increase in the Apo-B level [20]. Because Apo-A is a major carrier through which HDL-C transports lipids from the arterial wall to the liver for metabolism and subsequent excretion, Apo-A has an antiatherosclerotic effect [23]. In contrast, Apo-B is a major component of LDL-C and can promote atherosclerosis. The dysregulation of the ratio of Apo-A to Apo-B thus increases the risk of atherosclerosis [23]. The prevalence of obesity in PCOS patients is influenced by ethnicity [24]. Our present study showed that the proportion of obese Chinese PCOS patients is 26% while that of nonobese patients is 74% (when calculated by BMI), which indicates that most of Chinese PCOS patients are nonobese PCOS patients.

Previous studies have found that the prevalence of MS in PCOS patients is significantly higher than that in healthy controls. Dokras et al. showed that the prevalence of MS in 129 PCOS patients in the United States was 47.3% [12]. Santos et al. reported a prevalence of 27.0% in Portugal [25]. Hahn et al. reported a prevalence of 31.5% in Germany [26]. In Asian or Chinese populations, the prevalence of MS in PCOS patients was reported to range from 16.0% to 35.3% [27, 28]. The prevalence of MS in the PCOS patients in these previous studies is inconsistent; the inconsistency might be related to differences in genetic factors, race, diet, and lifestyle or to different MS diagnostic criteria. Based on the IDF MS definition, our present study showed that 20.50% of the 200 PCOS patients were diagnosed with MS. This prevalence was significantly higher than that in the control group.

Previous studies have shown that the risk of MS is increased in PCOS patients older than 40 years of age [29]. In fact, our present results showed that PCOS associated with MS is increasingly occurring in younger populations, and this observation is consistent with those in previous studies [30]. Therefore, diagnostic biomarkers for MS in PCOS patients should be identified, and screening for MS components, which can provide a scientific basis for early intervention, should be carried out as early as possible in PCOS patients of different ages and different BMIs.

Although the prevalence of MS in Chinese PCOS patients is relatively low compared with that in other ethnic populations, our present study showed that the occurrence of one or more MS components in PCOS patients was 71.0%, which indicates that Chinese PCOS patients are susceptible to MS. In particular, abdominal obesity and abnormal lipid metabolism were the most prevalent metabolic abnormalities in Chinese PCOS patients. Although the prevalence was still increased by as much as threefold compared to that in the control group, the incidence of hypertension and impaired fasting glucose in Chinese PCOS patients was relatively low. By a long-term follow-up of 786 PCOS patients, Wild et al. found that cardiovascular risk factors were increased significantly in PCOS patients, which is a finding consistent with our present results [31]. Compared with patients without any MS components in our present study, PCOS patients with four or more MS components had an increased prevalence of clinical cardiovascular disease of more than fivefold. Therefore, clinicians should devote more attention to the prevalence of MS components in PCOS patients.

According to the revised MS diagnostic criteria of the IDF, central obesity is a prerequisite for MS diagnosis. However, studies assessing the ability of MS components included in the diagnostic criteria to screen the risk of MS in PCOS patients have not been reported. In Caucasian PCOS patients, it has been reported that 58% of 200 patients with PCOS had reduced levels of HDL-C, while only 11% of the patients had increased levels of TG [32]. We also found in our present study that 51.0% of the 200 Chinese PCOS patients had reduced levels of HDL-C and 19.50% of these patients had increased levels of TG. Therefore, reduced levels of HDL-C may play a prominent role in the incidence of MS in PCOS patients [30]. Moreover, our present study showed that HDL-C and WC coupled with TG are the two relatively reliable diagnostic biomarkers for MS in Chinese patients with PCOS. These results indicate that early intervention to prevent the occurrence of MS should be performed in PCOS patients with a WC ≥ 80 cm once dyslipidemia (HDL-C < 1.29 mmol/L or TG ≥ 1.7 mmol/L) occurs. Namely, our results suggest that intervention should be initiated when both WC and TG or both WC and HDL-C are abnormal rather than waiting until MS occurs to initiate intervention. Previous multiple prospective studies have confirmed that HDL-C levels are significantly negatively correlated with the risk of cardiovascular disease. The mechanism underlying HDL-C function is that HDL-C is involved in reverse cholesterol transport, in which cholesterol ester is transferred from peripheral cells back to the liver to be metabolized. HDL-C also has anti-inflammatory and antioxidant functions, which play antiatherosclerotic roles [31]. Furthermore, as a new indicator for evaluating cardiovascular risk, the combination of WC and TG is closely related to the severity of metabolic disorders and coronary artery disease [33]. The rationale for combining WC with TG is that WC, which is used as an index to evaluate central obesity, can approximately estimate the degree of visceral adipose tissue accumulation and the fasting serum TG level can indirectly reflect the level of LDL-C. Simultaneous increases in WC and TG reflect an impaired ability of the body to rapidly remove and store excess triglycerides in subcutaneous adipose tissue, e.g., an impaired protective function of metabolic deposition. Therefore, the combination of WC and TG is closely related to the severity of metabolic disorders.

## 5. Conclusions

We conclude that the prevalence of MS and its component abnormalities is higher in PCOS patients than in controls, and within the PCOS patients, the occurrence of MS significantly increases with increasing age and BMI. Furthermore, increased WC coupled with decreased HDL-C and increased WC coupled with elevated TG may serve as simple and reliable diagnostic biomarkers for MS in PCOS patients in the Chinese population.

## Figures and Tables

**Table 1:** Characteristics of PCOS patients and healthy controls (*n* = 401).

Variable	Controls (*n* = 201)	PCOS (*n* = 200)	*Z* score	*P* value
Age (years)	26.0 (23.0–29.0)	27.0 (24.5–29.0)	−0.823	0.410
Weight (kg)	56.0 (50.0–60.50)	57.0 (50.0–64.80)	−1.148	0.251
BMI (kg/m^2^)	21.76 (20.31–23.85)	22.48 (20.09–25.10)	−1.334	0.182
WC (cm)	75.0 (70.0–79.50)	77.0 (71.25–84.0)	−3.088	**0.002**
WHR	0.86 (0.80–0.90)	0.86 (0.83–0.90)	−0.540	0.589
TT (ng/dL)	0.43 (0.30–0.52)	0.89 (0.75–1.12)	−15.065	**<0.001**
DHEAS (ng/dL)	198.20 (146.10–265.95)	279.90 (211.38–372.88)	−7.750	**<0.001**
TG (mmol/L)	0.76 (0.59–0.96)	1.06 (0.70–1.53)	−5.892	**<0.001**
TC (mmol/L)	4.16 (3.74–4.61)	4.69 (4.12–5.33)	−6.370	**<0.001**
HDL-C (mmol/L)	1.38 (122–1.60)	1.28 (1.10–1.54)	−3.025	**0.002**
LDL-C (mmol/L)	2.42 (2.12–2.82)	2.81 (2.35–3.36)	−6.056	**<0.001**
Apo-A (g/L)	1.17 (1.02–1.41)	1.12 (1.0–1.28)	−2.292	**0.022**
Apo-B (g/L)	0.65 (0.60–0.77)	0.78 (0.67–0.94)	−6.941	**<0.001**
SBP (mmHg)	114.0 (109.5–119.0)	115 (108.0–120.0)	−1.347	0.178
DBP (mmHg)	71.0 (65.0–77.0)	72.0 (67.0–80.0)	−2.433	**0.015**
FG (mmol/L)	4.51 (4.21–4.88)	4.69 (4.37–5.01)	−2.765	**0.006**
FI (mU/mL)	4.51 (3.13–5.73)	6.52 (4.29–10.93)	−6.611	**<0.001**
HOMA-IR	0.89 (0.62–1.25)	1.35 (0.86–2.33)	−6.626	**<0.001**
QUICK	0.39 (0.37–0.42)	0.37 (0.34–0.39)	−6.626	**<0.001**

Data are expressed as the median (25% and 75% interquartile ranges) values. The bold values represent differences that were considered statistically significant at *P* < 0.05.

**Table 2:** Prevalence of MS and MS components in PCOS patients (*n* = 401).

Variable	Controls (*n* = 201)	PCOS (*n* = 200)	*χ* ^2^	*P* value
Prevalence of MS, *n* (%)	6 (2.99)	41 (20.50)	29.723	**<0.001**
Occurrence of each component of MS, *n* (%)				
WC ≥ 80 cm	50 (24.88)	87 (43.50)	15.460	**<0.001**
TG > 1.7 mmol/L	10 (4.98)	39 (19.50)	19.718	**<0.001**
HDL-C < 1.29 mmol/L	57 (28.36)	102 (51.0)	21.477	**<0.001**
SBP ≥ 130 mmHg and/or DBP ≥ 85 mmHg	7 (3.48)	27 (13.50)	12.964	**<0.001**
FG > 5.6 mmol/L	3 (1.49)	11 (5.50)	4.778	**0.029**
Number of MS criteria, *n* (%)			47.876	**<0.001**
0	103 (51.24)	58 (29.0)		
1	73 (36.32)	69 (34.50)		
2	19 (9.45)	32 (16.0)		
3	6 (2.99)	33 (16.5)		
4	—	6 (3.0)		
5	—	2 (1.0)		

Data are expressed as the number (%) of patients. The bold values represent differences that were considered statistically significant at *P* < 0.05.

**Table 3:** Analysis of the occurrence of MS components in PCOS patients by age and BMI (*n* = 200).

Variable, *n* (%)	Age						BMI					
	<25.0 (*n* = 60)	25.0–29.9 (*n* = 93)	30.0–34.9 (*n* = 42)	≥35.0 (*n* = 5)	*χ* ^2^	*P* value	<18.5 (*n* = 19)	18.5–22.9 (*n* = 92)	23.0–24.9 (*n* = 37)	≥25.0 (*n* = 52)	*χ* ^2^	*P* value
WC ≥ 80 cm	23 (38.33)	39 (41.94)	22 (52.38)	3 (60.0)	2.393	0.122	0	11 (11.96)	26 (70.27)	50 (96.15)	113.58	**<0.001**
TG > 1.7 mmol/L	5 (8.33)	23 (24.73)	10 (23.81)	1 (20.0)	3.817	0.051	0	10 (10.87)	8 (21.62)	21 (40.38)	22.969	**<0.001**
HDL-C < 1.29 mmol/L	32 (53.3)	48 (51.6)	23 (54.8)	3 (60.0)	0.050	0.822	3 (15.8)	44 (47.8)	20 (54.1)	39 (75.0)	19.395	**<0.001**
SBP ≥ 130 mmHg and/or DBP ≥ 85 mmHg	10 (16.67)	12 (12.90)	4 (9.52)	1 (20.0)	0.597	0.440	1 (5.26)	6 (6.52)	4 (10.81)	16 (30.77)	15.432	**<0.001**
FG > 5.6 mmol/L	0	9 (9.68)	2 (4.76)	0	0.937	0.333	0	2 (2.17)	3 (8.11)	6 (11.54)	6.940	**0.008**

Data are expressed as the number (%) of patients. The bold values represent differences that were considered statistically significant at *P* < 0.05.

**Table 4:** Logistic analysis of the characteristics of PCOS patients with MS and without MS.

Variable, *n* (%)	No MS (*n* = 159)	MS (*n* = 41)	OR (95% CI)
Age			
<25.0	52 (32.7)	8 (19.5)	1.000
25.0–29.9	74 (46.5)	19 (46.3)	1.669 (0.679–4.101)
30.0–34.9	30 (18.9)	12 (29.3)	2.600 (0.955–7.075)
≥35.0	3 (1.9)	2 (4.9)	4.333 (0.624–30.090)
BMI (kg/m^2^)			
<18.5	19 (11.9)	0 (0.0)	—
18.5–22.9	88 (55.3)	4 (9.8)	1.000
23.0–24.9	27 (17.0)	10 (24.4)	**8.148** (**2.365**–**28.076**)
≥25.0	25 (15.7)	27 (65.9)	**23.760** (**7.596**–**74.303**)
WC (cm)			
<80	113 (71.1)	0 (0.0)	**—**
≥80	46 (28.9)	41 (100.0)	—
TG (mmol/L)			
≤1.7	148 (93.1)	13 (31.7)	1.000
>1.7	11 (6.9)	28 (68.3)	**28.979** (**11.796**–**71.193**)
HDL-C (mmol/L)			
≥1.29	90 (56.6)	4 (9.8)	1.000
<1.29	69 (43.4)	37 (90.2)	**12.065** (**4.105**–**35.465**)
Hypertension (mmHg)			
SBP < 130 and DBP < 85	152 (95.6)	21 (51.2)	1.000
SBP ≥ 130 or DBP ≥ 85	7 (4.4)	20 (48.8)	**20.680** (**7.807**–**54.783**)
FG (mmol/L)			
<5.6	155 (97.5)	34 (82.9)	1.000
≥5.6	4 (2.5)	7 (17.1)	**7.978** (**2.211**–**28.789**)

Data are expressed as the number (%) of patients. The bold values represent differences that were considered statistically significant at *P* < 0.05.

**Table 5:** Ability of the potential biomarkers to diagnose MS in PCOS patients (*n* = 200).

Biomarkers	PPV (%)	NPV (%)	Specificity (%)	Sensitivity (%)	Youden's index
WC	54.83	94.93	82.39	82.93	65.32
TG	69.23	91.30	92.45	65.85	58.30
BP	70.37	87.28	94.97	46.34	41.31
FG	63.64	82.01	97.48	17.07	21.41
WC + HDL-C	59.68	97.10	84.28	90.24	74.52
WC + TG	93.33	92.35	98.74	68.29	67.03
WC + BP	95.24	88.27	99.37	48.78	48.15
WC + FG	87.5	82.29	99.37	17.07	16.44

## Data Availability

The data used to support the findings of this study are available from the corresponding author upon request.

## Abbreviations

PCOS: Polycystic ovary syndrome
MS: Metabolic syndrome
BMI: Body mass index
TG: Triglycerides
HDL-C: High-density lipoprotein cholesterol
LDL-C: Low-density lipoprotein cholesterol
WC: Waist circumference
AUCs: The areas under the ROC curves
SBP: Systolic blood pressure
DBP: Diastolic blood pressure
FG: Fasting glucose
WHR: Waist-to-hip ratio
TT: Total testosterone
DHEAS: Dehydroepiandrosterone sulfate
FI: Fasting insulin
TC: Total cholesterol
Apo-A: Apolipoprotein A
PPV: Positive predictive value
NPV: Negative predictive value
HOMA-IR: Homeostasis model assessment of insulin resistance
QUICKI: Quantitative insulin sensitivity check index
OR: Odds ratio.

## Supplementary Materials

Supplementary Materials We provide the datasets of our present study (PCOS and control patients).
